# Variants in the *SNCA* Locus Are Associated With the Progression of Parkinson's Disease

**DOI:** 10.3389/fnagi.2019.00110

**Published:** 2019-05-21

**Authors:** Ningdi Luo, Yuanyuan Li, Mengyue Niu, Liche Zhou, Mengsha Yao, Lin Zhu, Guanyu Ye, Wenyan Kang, Jun Liu

**Affiliations:** ^1^Department of Neurology and Institute of Neurology, Ruijin Hospital Affiliated to Shanghai Jiaotong University School of Medicine, Shanghai, China; ^2^Ruijin Hospital North Affiliated to Shanghai Jiaotong University School of Medicine, Shanghai, China; ^3^CAS Center for Excellence in Brain Science and Intelligence Technology, Shanghai, China

**Keywords:** Parkinson's disease, RBD, SNCA, SNP, disease progression

## Abstract

**Background:** Genetic factors have a well-known influence on Parkinson's disease (PD) susceptibility; however, no previous studies have investigated the influence of *SNCA* mutations on the natural history of PD using a prospective follow-up study. The aim of this study was to assess the risk factors of variation of *SNCA* on the prognosis symptoms of PD patients.

**Methods:** Fifty PD patients were recruited with 38 v-PSG confirmed PD+RBD patients, and the median follow-up period was 30 months. All patients underwent a comprehensive clinical evaluation at baseline and follow-up, and six SNPs of *SNCA* (rs356165, rs3857053, rs1045722, rs894278, rs356186, and rs356219) were analyzed. Cox proportional hazards regression models and Kaplan–Meier plot analysis were used to assess the associations between the *SNCA* variation and the primary and secondary progression outcomes.

**Results:** Based on the clinical assessment, we found that hyposmia was substantially easier to aggravate. Regression analysis showed that patients with the T allele of rs1045722 and the G allele of rs356219 presented a 34 and 20% decreased risk of progression to the H-Y stage, respectively (*p* = 0.022; *p* = 0.005). While for rs894278, G allele patients showed a 47% decreased risk of olfactory dysfunction (*p* = 0.029). Further subgroup analysis showed that PD+RBD patients with rs356219/G exhibited a 30% and 20% decreased risk of progression on the H-Y stage and MoCA score (*p* = 0.038; *p* = 0.045).

**Conclusions:** Our results indicated that genetic variation in *SNCA* may contribute to variability natural progression of PD and could possibly be used as a prognostic marker.

## Introduction

Parkinson's disease (PD) is a multifactorial neurodegenerative disease associated with a combination of motor and non-motor features, while motor disability and cognitive decline may have poor influence on the quality of life in patients with PD (Zhang et al., 2005; Barnett, 2016; Chahine et al., 2016; Sveinbjornsdottir, 2016). The rates of clinical symptom progression and severity in PD are highly heterogeneous. Currently, the prognostic factors include gender, age at onset, disease subtype, and early cognitive status (Konno et al., 2016), while it remains largely unknown what factors indeed influence the long-term clinical progression and outcomes, especially in different subtypes of PD.

Previous studies have shown that genetic mutations play important roles in the pathogenesis of the sporadic form of PD, including *SNCA, Parkin, PINK1, DJ-1*, and *LRRK2*, in which *SNCA* is one of the most prominent hallmarks (Houlden and Singleton, 2012; Han et al., 2015; Tatura et al., 2016). For a better understanding of the progression of disease, a comprehensive analysis between clinical evaluations and genotypes in PD is necessary.

Rapid eye movement behavior disorder (RBD) is one of the dominant non-motor symptoms, which could appear before or after the motor symptoms. Multiple studies have confirmed that RBD is a risk factor for motor impairment and cognitive dysfunction (Postuma et al., 2012; Chahine et al., 2016; Li et al., 2017). Besides, PD patients encoded by the gene *SNCA* were several supportive evidence in RBD (Paul et al., 2018). In this study, we would investigate whether SNCA variants are associated with the development of the natural clinical evolution and relevant to RBD subtype. Thus, identifying prognostic markers that would influence disease progression would dramatically accelerate research into PD etiology and strengthen precision management of PD.

## Methods

### Participants

All study participants were recruited from the Department of Neurology, Ruijin Hospital affiliated with Shanghai JiaoTong University School of Medicine. At baseline, 50 PD participants were recruited to (1) be diagnosed according to the UK Brain Bank or current clinical diagnostic criteria by at least two movement disorders specialists (Hughes et al., 1992); (2) be 50 to 80 years old; (3) not have dementia or psychiatric disease; and (4) not have severe primary diseases, such as cardiac, liver, renal, and hematopoietic system diseases. Patients were divided into RBD (+) and RBD (–) groups according to the results of video polysomnography (v-PSG), based on the diagnostic criteria of the International Classification of Sleep Disorders (ISCD)-II criteria. In addition, all patients were designed to a follow-up after an average of 30 months. This study was approved by the ethics committee of Ruijin Hospital, Shanghai JiaoTong University School of Medicine. All participants or their guardians provided written informed consent.

### Demographics and Clinical Assessment

Disease stage was determined using the modified Hoehn-Yahr (H-Y) staging (Hoehn and Yahr, 2001), while the motor subscale of Unified Parkinson's Disease Rating Scale (UPDRS) was used to evaluate motor symptoms (Movement Disorder Society Task Force on Rating Scales for Parkinson's Disease., 2003). Moreover, a series of comprehensive questionnaires in addition to the evaluation of motor symptoms were also administered for every patient, including the Non-Motor Symptom Questionnaire (NMSQ), REM Sleep Behavior Disorder Screening Questionnaire (RBDSQ), Scale for Outcomes in PD-Autonomic (SCOPA-AUT), olfaction test (SS-16), Mini-Mental State Examination (MMSE), and Montreal Cognitive Assessment (MoCA). The NMSQ was used to evaluate non-motor symptoms, while the SCOPA-AUT was employed to assess autonomic dysfunction (Verbaan et al., 2007). An olfaction test (SS-16) was also performed to assess the olfactory function through a 16-item odor identification (Hummel et al., 1997; Krismer et al., 2017). The MMSE and MoCA were performed to evaluate the cognitive function resulting from PD. In addition, an overnight v-PSG examination was performed for each patient to confirm the diagnosis of RBD, meeting the ISCD-II.

### Genotyping

Previous studies have shown that *SNCA* rs356186 and rs356219 were associated with PD (Han et al., 2015), while rs356165, rs3857053, rs1045722, and rs894278 were associated with RBD (Li et al., 2017; Toffoli et al., 2017). Thus, we measured these six SNPs in the recruited patients (primers seen in Supplemental Table 1). Genomic DNA was extracted from lymphocytes of whole blood using the QIAamp DNA extraction kit (Qiagen, Valencia, CA, USA). The primers for sequences of SNP were designed by using Primer bank online design software and checked by the Shanghai BioSune Applied Biotechnology Company. Genotypes of the six loci were obtained directly by DNA sequencing run on a 3730xl DNA analyzer (Applied Biosystems, Foster City, CA, USA). The results were further analyzed by using the Chromas software (Version 2.6.5).

### Statistical Analysis

Continuous data were presented as the mean ± standard deviation (SD), while discontinuous data were presented as median values (quartile). Paired samples *t*-test was conducted for the comparison of the differences in continuous data, and the Mann–Whitney *U*-test was performed to compare the difference in ranked data (H-Y stage) between the patients at baseline and the last visit. To assess associations between the *SNCA* variation and progression outcomes, Cox proportional hazards regression models were used to estimate hazard ratios (HRs) with 95% confidence intervals (95% CI) adjusted for age and sex and Kaplan–Meier plot and log rank test to analyze disease risk. Categorical variables were compared with the χ^2^ or Fisher exact test to assess the difference between the RBD (+) and RBD (−) group at baseline. All tests were two-sided, and the threshold of significance was *p* < 0.05. All statistical analyses were performed using SPSS statistical software (Version 22.0).

## Results

### Demographic and Clinical Data

Fifty PD patients were enrolled in this study at baseline, and the cohort was followed up for a mean of 30 months for symptom progression. Demographics and clinical features of the baseline and follow-up data are listed in Table 1. The average age of the recruited PD patients at baseline was 64.6 years old, and 34 (68%) patients were male. The follow-up data showed a significantly higher H-Y stage, UPDRS scores of parts II and III, LEDD, and NMSQ than at the baseline (*p* ≤ 0.001, Table 1). Moreover, the aggravation of olfactory dysfunction was also found in the follow-up data (*p* = 0.022, Table 1). However, regarding the RBDSQ, SCOPA-AUT, MMSE, and MoCA scores, no significant difference was observed after the 30-month period.

**Table 1:** Demographic and clinical features of PD patients.

	**Baseline (*n* = 50)**	**Follow up (*n* = 50)**	***p* value**
Age, years	64.6 ± 5.4	/	/
Sex, *n* (%)			/
Male	34 (68)	/	/
Female	16 (32)	/	/
H-Y stage	1.5 (0.5)	2.0 (0.5)	**<0.001^a^**
UPDRS II	8.9 ± 4.2	13.1 ± 5.3	**<0.001^b^**
UPDRS III	16.6 ± 10.1	26.1 ± 12.1	**<0.001^b^**
LEDD (mg/day)	270.4 ± 286.9	433.0 ± 235.6	**<0.001^b^**
NMSQ	8.6 ± 4.2	10.6 ± 4.7	**0.001^b^**
SS-16	8.2 ± 3.3	7.3 ± 3.4	**0.022^b^**
RBDSQ	5.6 ± 3.3	6.4 ± 3.7	0.070^b^
SCOPA-AUT	13.0 ± 8.7	15.4 ± 8.7	0.056^b^
MMSE	28.1 ± 1.9	28.0 ± 2.1	0.584^b^
MoCA	25.3 ± 3.3	24.7 ± 4.2	0.194^b^

*PD, Parkinson's disease; H-Y stage, Hoehn-Yahr stage; UPDRS, Unified PD Rating Scale; LEDD, levodopa equivalent daily doses; NMSQ, Non-Motor Symptom Questionnaire; SS-16, Sniffin' Sticks 16-item test; RBDSQ, REM Sleep Behavior Disorder Screening Questionnaire; SCOPA-AUT, Scale for Outcomes in PD-Autonomic; MoCA, Montreal Cognitive Assessment*.

a *p-value was calculated from Mann–Whitney U-test*.

b *p-values were calculated from paired samples t-test*.

*The bold emphasis in the p column means p < 0.05 with statistically significant*.

### Estimated Primary Clinical Outcomes in PD Patients With SNCA Variation

Changes >2.5 to 5.2 points on the UPDRS-III motor score represent clinically meaningful differences (Shulman et al., 2010). Thus, we defined a rapid motor progression, based on the mean 2.6 years of follow-up, as a 10-point increase of the UPDRS-III score (mean of 4 points per year) as a previous study performed (Paul et al., 2018). For the other motor indexes, including the UPDRS-II, H-Y stage, and LEDD, and non-motor symptoms, no significantly changed point has been defined. As a 10-point increase of the UPDRS-III score was exactly the average variation between the baseline and follow-up data, we thus specified the mean deviation from the baseline to first follow-up examinations as the cutoff value for further analyses of clinical outcomes, as shown in Table 2.

**Table 2:** Estimated HRs for clinical outcomes in PD patients of *SNCA* variation^a^.

**Outcome**	**rs356219 (G dominant) (*****n*** **=** **40)**		**rs1045722 (T dominant) (*****n*** **=** **29)**		**rs894278 (G dominant) (*****n*** **=** **20)**	
	**HR (95% CI)**	***p***	**HR (95% CI)**	***p***	**HR (95% CI)**	***p***
**PRIMARY ENDPOINTS**						
Time to HY 0.5-point increase	0.20 (0.07–0.61)	**0.005**	0.34 (0.14–0.86)	**0.022**	0.57 (0.27–1.25)	0.162
Time to UPDRS II 5-point increase	0.54 (0.15–1.98)	0.356	0.98 (0.42–2.27)	0.959	0.88 (0.38–2.04)	0.778
Time to UPDRS III 10-point increase	0.49 (0.1–2.44)	0.386	0.42 (0.15–1.20)	0.106	0.70 (0.27–1.85)	0.481
Time to LEDD 162-point increase	0.30 (0.79–1.10)	0.070	0.55 (0.20–1.51)	0.245	0.70 (0.29–1.67)	0.422
**SECONDARY ENDPOINTS**						
Time to NMSQ 2-point increase	0.54 (0.18–1.62)	0.268	0.67 (0.32–1.41)	0.291	0.88 (0.43–1.81)	0.730
Time to SS-16 1-point decrease	0.32 (0.12–0.85)	**0.021**	0.65 (0.31–1.34)	0.241	0.47 (0.23–0.92)	**0.029**
Time to SCOPA 2-point increase	0.80 (0.18–3.62)	0.775	0.93 (0.37–2.33)	0.877	0.56 (0.35–2.08)	0.722
Time to MoCA 1-point decrease	0.18 (0.05–0.61)	**0.006**	0.52 (0.21–1.32)	0.170	0.53 (0.27–1.27)	0.151

*PD, Parkinson's disease; H-Y stage, Hoehn-Yahr stage; UPDRS, Unified PD Rating Scale; LEDD, levodopa equivalent daily doses; NMSQ, Non-Motor Symptom Questionnaire; SS-16, Sniffin' Sticks 16-item test; RBDSQ, REM Sleep Behavior Disorder Screening Questionnaire; SCOPA-AUT, Scale for Outcomes in PD-Autonomic; MoCA, Montreal Cognitive Assessment; HR, hazard ratio; 95% CI, 95% confidence interval*.

a *Cox proportional hazards regression models were used to estimate HRs, with 95% CI adjusted for age and sex. P-values were calculated using the Wald χ^2^-test*.

*The bold emphasis in the p column means p < 0.05 with statistically significant*.

**Figure 1:**
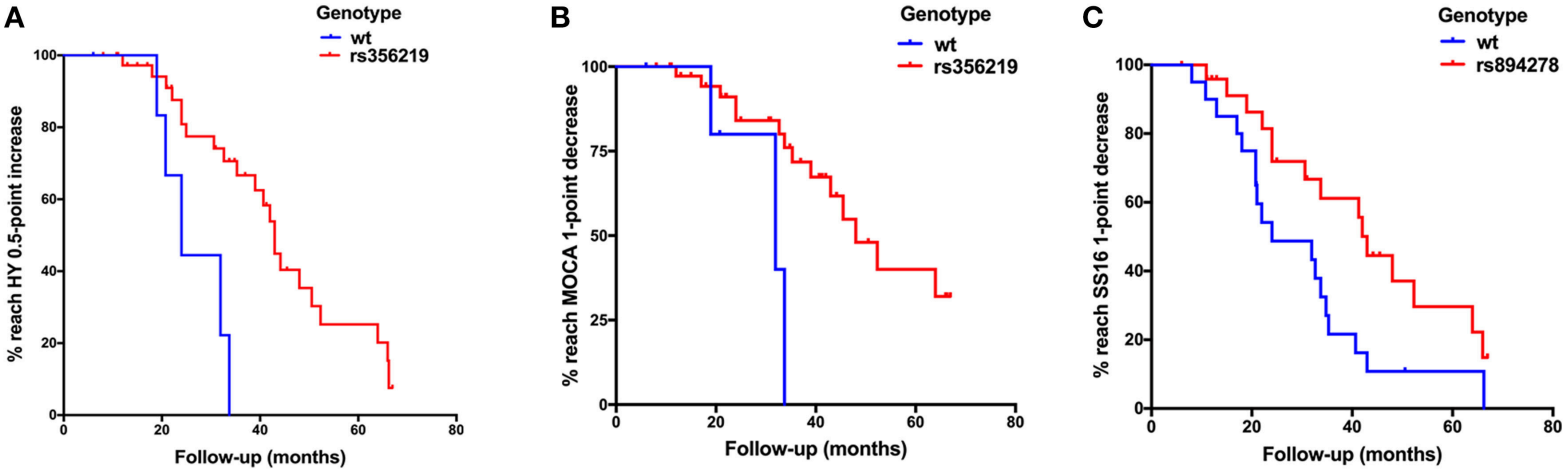
**(A)** Kaplan–Meier plot of H-Y stage to reach a 0.5-point increase in patients with G dominant models of rs356219 appeared a slower progression than *WT* genotype (*p* = 0.002). **(B)** Kaplan–Meier plot of Montreal Cognitive Assessment (MoCA) to reach a 1-point decrease in patients with G dominant models of rs356219 appeared a slower progression than *WT* genotype (*p* = 0.002). **(C)** Kaplan–Meier plot of SS-16 to reach a 1-point decrease in patients with G dominant models of rs894278 appeared a slower progression than *WT* genotype (*p* = 0.024).

The progression of motor symptoms was set as the primary endpoints, including the H-Y stage, UPDRS II, UPDRS III, and LEED. For the H-Y stages, we defined a change of 0.5 points as disease progression. The results showed that the patients with the G allele of rs356219 and the T allele of rs1045722 of *SNCA* presented a 20% and 34% decreased risk of progression on the H-Y stage, respectively (*p* = 0.005, 95% CI: 0.07–0.61; *p* = 0.022, 95% CI: 0.14–0.86, Table 2). The Kaplan–Meier analysis also indicated that, compared to the patients with the *WT* genotype, the patients with G dominant models of rs356219 exhibited a longer progression-free-survival (PFS) time to reach a 0.5-point increase of the H-Y stage (42 vs. 24 months, *p* = 0.002, Figure 1A). For the UPDRS, we defined a 5-point change for the UPDRS II and a 10-point change for the UPDRS III as the progression index. Moreover, for the LEDD, we defined the subtraction of the mean 162 point as the cutoff value. However, no statistical significance was identified among rs356219, rs1045722, and rs894278 for the UPDRS or LEDD.

### Estimated Secondary Clinical Outcomes in PD Patients With SNCA Variation

Non-motor symptom aggravation was defined as the secondary endpoint, including the NMSQ, SCOPA-AUT, SS-16, and MoCA. We defined a two-point increase as non-motor symptom progression, a two-point increase as autonomic nervous disorder aggravation, a one-point decrease as olfactory decline, and a one-point decrease as cognitive decline. We found that the patients carrying the G allele of rs356219 showed a 32% decreased risk of olfactory dysfunction (*p* = 0.021, 95% CI: 0.12–0.85, Table 2), and the patients with the G allele of rs894278 presented a 47% decreased risk of olfactory dysfunction (*p* = 0.029, 95% CI: 0.23–0.92, Table 2). The Kaplan–Meier analysis also indicated a longer median PFS time to the SS-16 one-point decrease for the patients with the G allele of rs894278 (42 vs. 24 months, *p* = 0.024, Figure 1B). Furthermore, we found that the patients carrying the G allele of rs356219 presented an 18% decreased risk of suffering from cognitive decline (*p* = 0.005, 95% CI: 0.05–0.61, Table 2). The Kaplan–Meier analysis indicated median progression-free survival times of 45.5 months in the group of the G allele of rs356219 and 31.9 months in the remaining population (*p* = 0.002; Figure 1C).

### Subgroup Analysis of Progression of rs356219 of SNCA in PD Patients With RBD

As reported, PD patients with RBD typically presented worse disease progress and cognitive dysfunction (Chahine et al., 2016), and we further analyzed the clinical outcomes in a subgroup of PD patients with RBD or without RBD. However, no significant difference was identified on the H-Y stage, MMSE, or MoCA scores compared to the baseline (Table 3). Given that rs356219 was associated with cognitive function in Table 2, we further investigated the potential predictors of rs356219/G in the RBD subgroup. The results showed that PD+RBD patients with rs356219/G of *SNCA* presented a 30% and 20% decreased risk of progression on the H-Y stage and MoCA score (*p* = 0.038, 95% CI: 0.08–0.93; *p* = 0.045, 95% CI: 0.63–0.97, Table 3).

**Table 3:** Clinical outcomes in the RBD subgroup of PD patients.

	**RBD (–) (*n* = 12)**	**RBD (+) (*n* = 38)**	***P***	**RBD (+) (*n* = 38)**	**rs356219/G (*****n*** **=** **30)**	
					**HR (95% CI)**	***p***
HY	1.0 (1.0)	1.5 (0.5)	0.373^a^	Time to HY 0.5-point increase	0.3 (0.08–0.93)	**0.038**^b^
MoCA	24.7 ± 3.5	25.5 ± 3.2	0.476^a^	Time to MoCA 1-point decrease	0.2 (0.63–0.97)	**0.045**^b^
MMSE	28.1 ± 1.7	28.0 ± 2.4	0.629^a^	Time to MMSE 1-point decrease	0.3 (0.07–1.71)	0.190^b^

*RBD, REM sleep behavior disorder; rs356219/A, A allele of rs356219 of SNCA; H-Y stage, Hoehn-Yahr stage; UPDRS, Unified PD Rating Scale; LEDD, levodopa equivalent daily doses; NMSQ, non-motor symptom questionnaire; SS-16, Sniffin' Sticks 16-item test; RBDSQ, REM Sleep Behavior Disorder Screening Questionnaire; SCOPA-AUT, Scale for Outcomes in PD-Autonomic; MoCA, Montreal Cognitive Assessment; HR, hazard ratio; 95% CI, 95% confidence interval*.

a *p-value was calculated using the χ^2^ or Fisher exact test*.

b *Cox proportional hazards regression models were used to estimate HRs, with 95% CI adjusted for age and sex. P-values were calculated using the Wald χ^2^-test*.

*The bold emphasis in the p column means p < 0.05 with statistically significant*.

## Discussion

Up to now, few studies have focused on the correlation between clinical evaluation of the progression of PD and genotypes. In our current study, we found higher H-Y stage, UPDRS scores of parts II and III, LEDD, and NMSQ, and worse olfactory function in the 30-month progression of PD. *SNCA* rs894278/G and rs356219/G were associated with slower olfactory progression. Furthermore, we first found that rs356219/G reduced the risk of disease progression and cognitive impairment, even in the subgroup of PD+RBD. In addition, we first pointed out that *SNCA* rs1045722/T decreased disease progression.

A previous longitudinal population-based cohort study of 285 PD patients of European ancestry set a faster progression to H-Y stage 3 or a change of one stage as the point to explore motor progression in PD (Paul et al., 2018). This variability may be attributed to factors such as sample size, follow-up duration, and patient demographics. Moreover, it was reported that changes of 2.5 to 5.2 points on the UPDRS-III represent clinically meaningful differences (Shulman et al., 2010). We thus defined a rapid motor progression, based on the mean 2.6 years of follow-up, as a 10-point increase of the UPDRS-III score (mean of 4 points per year) as a previous study performed (Paul et al., 2018). Given that no dominant changed point has been defined for other indexes and a 10-point increase of the UPDRS-III score was exactly the average variation between the baseline and follow-up data, we specified the mean deviation from the baseline to first follow-up examinations as the cutoff value.

Further, we evaluated six SNPs of *SNCA* to identify its influence on the clinical progression. Based on our analysis, we demonstrated that patients carrying rs894278/G were prone to suffer hyposmia slower. In our previous studies, rs894278/G seems to be a risk allele, since PD patients were more likely to suffer from hyposmia (Chen et al., 2015; Li et al., 2017). However, as previous cross-sectional studies have shown, olfactory dysfunction was not always stable and it did not deteriorate in a linear manner during disease duration (Del Tredici et al., 2002; Braak et al., 2003). Moreover, Michelle E. Fullard's team explained that olfactory dysfunction might just be due to early pathology of the olfactory bulb; thus, it had no further impact on aggravation as the disease progresses deeper and Lewy bodies further deposit (Fullard et al., 2017). Therefore, olfaction score was not a reliable representative of disease progression for individuals. In addition, we found that patients with rs356219/G tend to retain better olfactory function, and it was the first time to investigate the influence of this SNP on hyposmia. In general, further research is required to determine the role of rs894278 and rs356219 in the hyposmia of PD patients.

As for cognitive function, previous studies have reported that ~80% of patients are expected to develop dementia after 10 years from the diagnosis of PD, and 20–30% of patients exhibit impairment that do not meet the criteria for dementia (Hoops et al., 2009; Aarsland and Kurz, 2010). The MMSE was the most widely used instrument used for cognitive assessment, while several independent studies have shown that the MoCA has greater specificity and accuracy than the MMSE (Hoops et al., 2009).

Previous studies have shown the rs356219/G was a significant risk for PD in different populations (Mata et al., 2010; Han et al., 2015; Emelyanov et al., 2018). It has been found that PD patients with rs356219/G had earlier onset age (Brockmann et al., 2013) and worse cognitive function (Campelo et al., 2017), probably affected by the distinction of *SNCA* expression in brain regions, blood, and plasma (Lesage and Brice, 2012; Brockmann et al., 2013; Burciu et al., 2018), whereas it was firstly shown in our study that rs356219/G reduces the risk of disease progression and cognitive impairment, which was not contradictory with previous studies. Previous studies mainly explored the risk genotypes of PD, while we focused on the disease progression of PD patients. In other words, populations with the G allele of rs356219 presented with higher susceptibility of PD but are more likely to have a relative slower disease progression. Simultaneously, sample limitation, race, or environmental factors are needed to be taken into account.

RBD was a representational prodromal condition of PD, and prospective studies have declared that it would exceed the conversion rate to PD (Gan-Or et al., 2015) and could predict the non–tremor-predominant subtype (Postuma et al., 2015). A previous study has shown that RBD may be associated with at least a subset of PD-associated genes in a 56 RBD cohort (Gan-Or et al., 2015). In our study, however, there was no significant difference on H-Y stage, MMSE, or MoCA scores compared to the baseline in patients with and without RBD. Thus, we further investigated whether there was a difference between the variant of rs356219 loci in the RBD subgroup (*n* = 38). The results showed that patients with rs356219/G experienced a slower motor progression and cognitive decline, which meant that rs356219/G was a protective factor for PD+RBD patients. In addition, our results firstly indicated that patients with rs1045722/T of *SNCA* appear to exhibit slower motor progression. This finding was indirectly in line with the self-reliant research that indicated that the A allele of rs1045722 was more frequent in PD patients than in iRBD (Toffoli et al., 2017). More research is required to determine the role of this SNP in the development of PD.

To date, levodopa remains the mainstay of treatment for PD, and higher doses of levodopa and other drugs may potentially produce better symptomatic control, but more motor complications (Tomlinson et al., 2010). For this reason, it would be meaningful to consider whether genetic effects may also be related to levodopa responsiveness so as to guide neurologists to make individual treatment strategies for PD patients. In our current study, however, no statistical contribution of the genotypes to LEDD progression was found, mainly because our patients were allowed to take PD medications during the entire examination process. Moreover, a longer follow-up period is needed to further verify our results.

In summary, we found that the variations of *SNCA* could contribute to variability in the natural progression of PD and could possibly be used as a prognostic marker. The limitation of this study is the small sample size; however, it still provides a cue that the genotype may affect the phenotype during PD progression. This study would help us to deeply explore the disease progression of PD and thus to strengthen precision management and contribute to further treatment. The mechanisms of how these variants influenced the pathogenesis and progression of PD are worth exploring in future studies.

## Ethics Statement

This study was approved by the ethics committee of Ruijin Hospital, Shanghai JiaoTong University School of Medicine.

## Author Contributions

JL and WK conceived and supervised the project and contributed to patients' recruitment. NL, YL, JL, and WK drafted the manuscript. NL and YL performed data management and statistical analyses. NL, YL, MN, LZho, MY, LZhu, and GY contributed to neurological assessment and sample collection of enrolled subjects. All authors read and approved the final version of the manuscript.

### Conflict of Interest Statement

The authors declare that the research was conducted in the absence of any commercial or financial relationships that could be construed as a potential conflict of interest.

## Abbreviations

H-Y stage: Hoehn-Yahr stage
PD: Parkinson's disease
REM: rapid eye movement
RBD: REM behavior disorder
NMS: non-motor symptoms
NMSQ: Non-Motor Symptom Questionnaire
UPDRS: Unified Parkinson's Disease Rating Scale
LEDD: levodopa equivalent daily doses
RBDSQ: RBD Screening Questionnaire
SCOPA-AUT: Scale for Outcomes in Parkinson Disease-Autonomic
SS-16: Sniffin' Sticks 16-item test
MMSE: Mini-Mental State Examination
MoCA: Montreal Cognitive Assessment
v-PSG: video polysomnography
ISCD: International Classification of Sleep Disorder
DAT: dopamine transporter.
