# Supplementary material for: Variants in the SNCA Locus Are Associated With the Progression of Parkinson's Disease
Source: Front Aging Neurosci. 2019 May 21;11:110. doi: 10.3389/fnagi.2019.00110 (PMC6562243; doi:10.3389/fnagi.2019.00110)
Supplement: Supplementary file 1 [file Table_1.DOCX]

**Table 1 Primers for Upstream and Downstream Sequences of SNP**

| SNP | Forward Primers (5'-3') | Reverse Primers (5'-3') |
| --- | --- | --- |
| rs356186 | GGCCATTACTATCAAGCATCTG | TGTAACTGTGGCTGCAGACC |
| rs3857053 | ATTGATCCTCAGGCCACTTG | GAAGGTTAGAAAGTGGCGGTTA |
| rs1045722 | ATTGATCCTCAGGCCACTTG | GAAGGTTAGAAAGTGGCGGTTA |
| rs894278 | GATCAGCCAGGCACGTTTAG | AGCTGTAAAATTGGCTGATGAA |
| rs356165 | TGTCAGAAAGGTACAGCATTCACA | CCTGAAGCAACACTGCCAGA |
| rs356219 | GAGGGCTCAAAAACGTAATCAG | CCAACATACGCTTGAAGCATAA |
